# Assessment of water resource security in karst area of Guizhou Province, China

**DOI:** 10.1038/s41598-021-87066-5

**Published:** 2021-04-07

**Authors:** Liying Liu

**Affiliations:** 1 College of Mathematics and Statistics, Chongqing Technology and Business University, Chongqing, 400067 China; 2 Chongqing Key Laboratory of Social Economy and Applied Statistics, Chongqing, 400067 China

**Keywords:** Ecology, Environmental sciences

## Abstract

This paper presents the assessment of water resource security in the Guizhou karst area, China. A mean impact value and back-propagation (MIV-BP) neural network was used to understand the influencing factors. Thirty-one indices involving five aspects, the water quality subsystem, water quantity subsystem, engineering water shortage subsystem, water resource vulnerability subsystem, and water resource carrying capacity subsystem, were selected to establish an evaluation index of water resource security. In addition, a genetic algorithm and back-propagation (GA-BP) neural network was constructed to assess the water resource security of Guizhou Province from 2001 to 2015. The results show that water resource security in Guizhou was at a moderate warning level from 2001 to 2006 and a critical safety level from 2007 to 2015, except in 2011 when a moderate warning level was reached. For protection and management of water resources in a karst area, the modes of development and utilization of water resources must be thoroughly understood, along with the impact of engineering water shortage. These results are a meaningful contribution to regional ecological restoration and socio-economic development and can promote better practices for future planning.

## Introduction

The concept of water resource security emerged in the late 1990s; Water has been widely regarded as the most essential natural resource^1^. Water resource security is the capacity of a population to safeguard sustainable access to adequate quantities of water of an acceptable quality to sustain human livelihoods, well-being, and socio-economic development; and to preserve ecosystems in a climate of peace and political stability^2^. One of the major concepts of sustainable development goals is water security, which is the basic element of the global goal regarding water. Water resource security can be seen as the ability to provide an acceptable quantity and quality of water for human health, survival, and economic activity while keeping water-related risks within acceptable levels for human, environmental, and economic needs. It is difficult to achieve sustainable development if water resources are not secure^3^. Water resource security has became one of the international research spotlight in recently years^4–6^.

Regional water resource security should ensure the water resources demand of human, economy, ecology, society and environment^7^. The pressure, status, and response framework usually allows the assessment of water resource security^8^. In karst areas, water conservancy project can increase resilience to rainfall variability, and it is possible to enhance the economic development and also to mitigate some expected impacts of climate change^9^. In recently years, some water resource security assessment was emerged by considering climate change and human activity. It is found that groundwater level is negatively correlated with groundwater exploitation, but positively correlated with rainfall^10^. The climate periodicity should be considered in groundwater management^11^. Climate change will directly or indirectly affect the quality of groundwater^12^. Dimki believes that climate changes and human water demand**s** compound the problem, and preventing excessive exploitation of karst springs is a measure to prevent water shortages^13^. Climate simulations project a strong increase in temperature and a decrease of precipitation in many karst regions in the world over the next decades. Despite this potentially bleak future, few studies specifically quantify the impact of climate change on karst water resources^14^. Although progress had been made to promote the water resource security, there is still no satisfactory definition that accepted by most researchers. With climate change and human activities, water resource systems are facing greater complexity and uncertainty, and water environment systems are extremely vulnerable to impacts and damage, especially in karst areas. Karst areas have special hydrogeological characteristics and are easily affected by human activities, climate and land use changes, and other factors; Recovery can be difficult^15,16^. Protection and management of water resources in karst areas must be thoroughly understood, along with the impact of human activity at a regional level^17^. Therefore, different water resource security research initiatives are important for understanding the nature of the resources as well for the protection, effective management, and sustainable development of water resource systems.

Water resource systems are complex nonlinear systems with obvious randomness and uncertainty. With the influence of climate change and human activities, the characterization of water resource security becomes increasingly difficult. Therefore, the assessment of water resource security needs to be further developed and improved. Over the past several years, some studies have turned to artificial neural network (ANN) models to develop new means of assessing environmental variables^18^. ANN models have become a popular tool for environmental modeling because of their non-linearity and the fact that they perform well with noisy and limited data sets^19,20^. Several recent studies have illustrated the value of ANN models in the assessments of the quality of the aquatic environment^21,22^, water quality^23,24^, land ecological security^25^, carrying capacity of the aquatic environment^26^, and karst groundwater management^27^. However, despite the recognition of the importance of water resource security in karst areas and the value of ANN models for this purpose, there is no comprehensive protocol for the development of water resource security in karst whereby: (i) all components of the karst water environment and their interactions are accounted for; (ii) both the temporal and spatial dimensions are considered; and (iii) an ANN model is established to select evaluation indices, in which subjective and objective analysis are combined.

The GA optimizes the initial weights and thresholds of BP neural network to achieve the global optimization . This method can optimize the input variables to improve the accuracy of forecasting. GA-BP have been widely used in various fields, achieved good results, and become important intelligent algorithms^28,29^, and there is no exception in the water science field.

Therefore, the present study aimed to assess water resource security in a karst area using a GA-BP neural network and a time series from the Guizhou karst area from 2001 to 2015. The results will contribute to regional ecological restoration and socio-economic development.

## Study area

**Figure 1:**
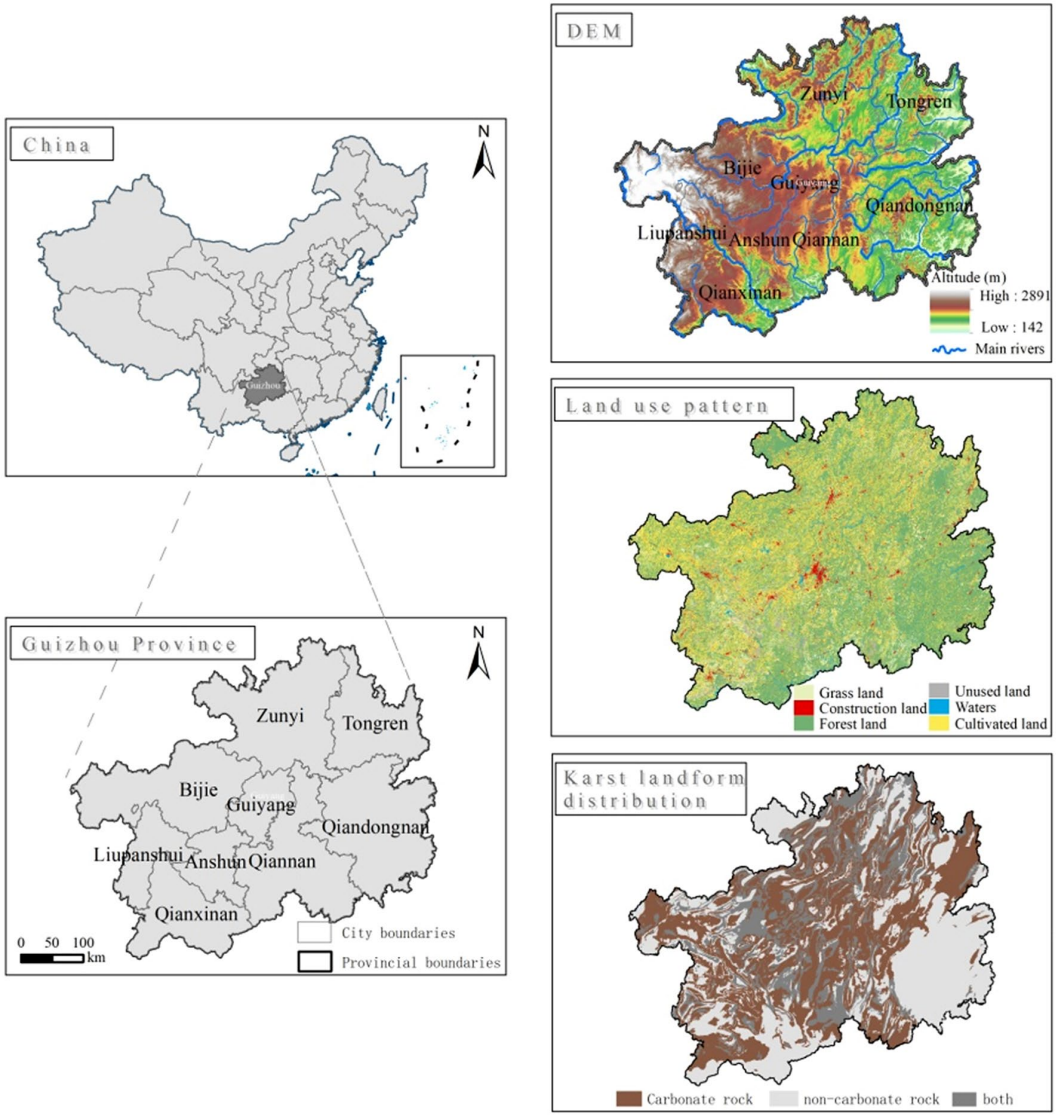
Overview of the study area. The study area map was created by ArcMap 10.6(http://www.esri.com/index.html) , using the data from CAS (http://www.csdb.cn/) .

Guizhou Province is located in the eastern part of the Yungui Plateau, the geography inclines downward from west to east, with an average elevation of 1100 m, longitudes of 103° 36′–109° 35′ E, and latitudes of 24° 37′–29° 13′ N (Fig. 1). Its area is 176,167 km^2^, of which the karst landform area covers 109,000 km^2^, accounting for 61.9% of the total. It is one of the most concentrated karst areas in the world, with the largest area of contiguous exposed carbonate rocks and intensively developed karst^30^. The main climate is sub-tropical moist monsoon climate. Water resources are abundant in Guizhou Province, with annual average precipitation of 1100–1300 mm, but this precipitation is unevenly distributed in space and time. May–August precipitation accounts for 85.1% of the annual total, and the rainy season is primarily during June and July. In addition, runoff and water resources in Guizhou Province have decreased, with significant declines in spring and autumn but insignificant increases in summer and winter over the past 50 years^31^. The annual consumption of water is about 10 billion m^3^, about 90% of which is from surface water supply. About 50% of water consumption is used for agriculture, about 30% for industry, and no more than 1% for ecological purposes. It is difficult to develop water resources in Guizhou, and the efficiency of water use is low.

## Materials and methodology

Water resource security is regarded as the integration of economy, society, and ecology, which interact in many complex ways. Therefore, water resource security must be assessed as a cohesive system. Its importance lies in ensuring a steady water supply for economic, societal, and ecological purposes. Its evaluation can start with evaluation of water conservation objectives, environmental vulnerability, and the ability to deliver services. In addition, human activities such as the regulation, supplementation, and enhancement of water resources via water conservancy projects have greatly influenced the variability and availability of water resources. Therefore, a framework was developed to examine water resource security based on five aspects, including a water quality subsystem, water quantity subsystem, engineering water shortage subsystem, water resource vulnerability subsystem, and water resource carrying capacity subsystem, which are characterized by 42 indices (Table 1).

**Table 1:** Primary evaluation index system for water resource security in the karst area.

Target	Index	Index meaning
Water quality security	*X*_1_ Treatment rate of urban waste water (%)	Reflects water quality
	*X*_2_ Qualifying rate of the water quality of rivers (%)	Reflects water quality of main rivers
	*X*_3_ Qualifying rate of water environment function zones (%)	Reflects the effectiveness of water resource management
	*X*_4_ Number of water pollution accidents (times)	Reflects the degree of environmental pollution caused by human activities
	*X*_5_ Discharge intensity of Chemical Oxygen Demand (COD) in industrial waste water (10^4^ t)	Reflects the degree of environmental pollution caused by economic development
	*X*_6_ Qualifying rate of industrial waste water (%)	Reflects water pollution treatment capacity
	*X*_7_ Comprehensive utilization rate of industrial solid waste (%)	Reflects water pollution treatment capacity
	*X*_8_ Fertilizer use per unit of cultivated land (kg/hm^2^ a)	Reflects the impact of agriculture on water quality
	*X*_9_ Total discharge of waste water (million tons)	Reflects the effectiveness of water resource management
Water quantity security	*X*_10_ Average annual rainfall (mm)	Reflects the status of water resources in the region
	*X*_11_ Groundwater resources per unit area (10^4^ m^3^/km^2^)	Reflects the richness of groundwater resources
	*X*_12_ Water resources per capita (m^3^/person)	Reflects the state of per capita water resources
	*X*_13_ Surface water resources per unit area (10^4^ m^3^/km^2^)	Reflects the state of water resources for each acre
	*X*_14_ Runoff depth (mm)	Reflects the status of water resources in the basin
	*X*_15_ Difference in water volume between entry and exit (10^8^ m^3^)	Reflects the balance of surface water
	*X*_16_ Water production modulus (10^4^ m^3^/km^2^)	Reflects the relative amount of water resources
	*X*_17_Water penetration rate (%)	Reflects the safety of drinking water
Engineering water shortage security	*X*_18_ Proportion of water supply for water lifting and diversion projects (%)	Reflects the difficulty of water resources development and utilization in karst areas
	*X*_19_ Storage rate of large and medium reservoirs (%)	Reflects the main water conservancy project storage capacity and efficiency
	*X*_20_ Satisfaction rate of farmland irrigation facilities (%)	Reflects the degree of protection of agricultural water use by farmland water conservancy facilities
	*X*_21_ Density of large and medium-sized reservoirs(per10^4^km^2^)	Reflects the ability of the engineering water supply
	*X*_22_ Ratio of environment and water conservancy personnel (%)	Reflects the capacity of water resources management
	*X*_23_ Ratio of effective irrigation area to cultivated land area (%)	Reflects the situation of farmland water conservancy construction
	*X*_24_ Water investment as a proportion of Gross Domestic Product (GDP) (%)	Reflects the adequacy of management funds
Water resources vulnerability security	*X*_25_ Soil erosion area ratio (%)	Reflects the state of soil erosion
	*X*_26_ Number of karst disasters (times)	Reflects the negative effects of water resources and the ecological environment
	*X*_27_ Sediment transport modulus (t/km^2^)	Reflects the state of soil erosion
	*X*_28_ Vegetation coverage (%)	Reflects the impact on surface water storage capacity
	*X*_29_ The ratio of moderate or severe rocky desertification area (%)	Reflects soil erosion status
	*X*_30_ Ecological water use rate (%)	Reflects the status of water resource carrying capacity
	*X*_31_ Per capita food production (kg)	Reflects the proportion of agricultural production
	*X*_32_ Flood and drought losses as a proportion of GDP (%)	Reflects the degree of negative effects of coupling between water resources and economic development
	*X*_33_ Urbanization rate (%)	Reflects the water pressure from regional development
Water resources carrying capacity security	*X*_34_ Water use efficiency (%)	Reflects the degree of water resource development
	*X*_35_ Degree of development and utilization of groundwater (%)	An important indicator of water supply capacity
	*X*_36_ Degree of development and utilization of surface water (%)	Reflects the sustainable utilization of water resources
	*X*_37_ Groundwater supply ratio (%)	Reflects the supply structure of water resources
	*X*_38_ Agricultural water use rate (%)	Reflects pressure from agricultural production on water resources
	*X*_39_ Water consumption per ten thousand yuan GDP (m^3^/ten thousand yuan)	Reflects the level of water consumption
	*X*_40_ Water consumption per ten thousand yuan industrial output (m^3^/ten thousand yuan)	Reflects the pressure of industrial water use on the quantity of water resources
	*X*_41_ Water consumption per ten thousand yuan agricultural output value (m^3^/ten thousand yuan)	Reflects the pressure of agricultural water use on the quantity of water resources
	*X*_42_ Irrigation water consumption per unit area (m^3^/ha)	Reflects the pressure of agricultural water use on the quantity of water resources

### Construction of the MIV-BP model for variable selection

Screening variables with significant features as the input data of the network is an important step in assessment of water resource security. It is necessary to select the most representative variables from the primary indices and to remove those that are not significant. For the neural network variables, mean impact value (MIV) screening can be considered one of the best algorithms to screen characteristic variables^32^.In this work, by combining a BP neural network with the MIV algorithm, the main influencing factors of water resource security were analyzed to establish the water resource security evaluation system.

**Figure 2:**
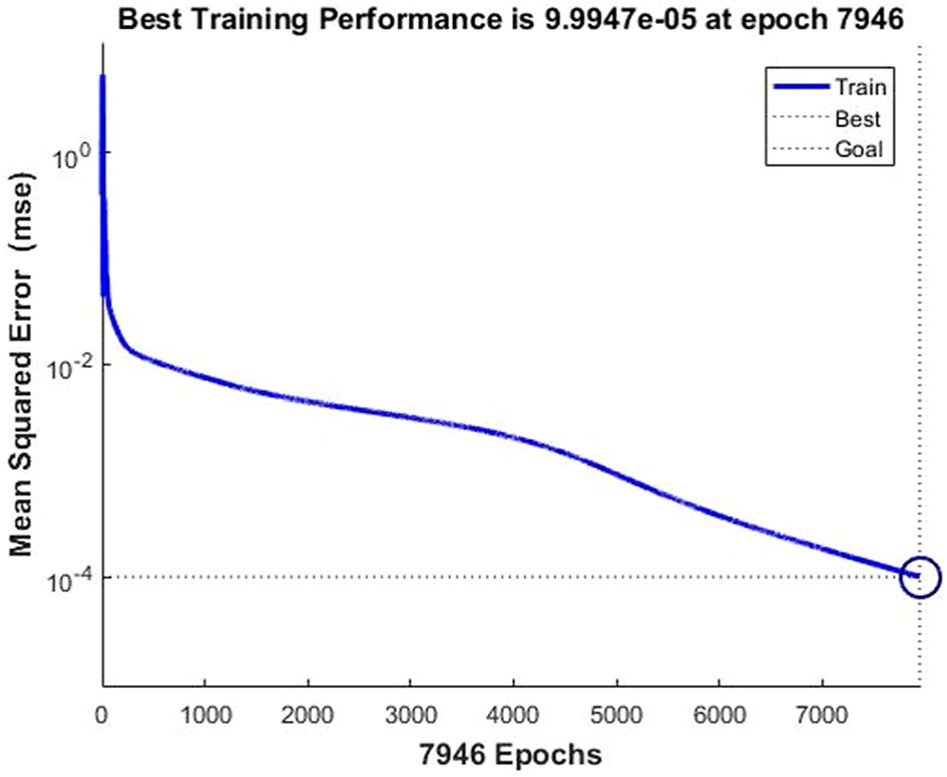
Effect of neural network training.

**Figure 3:**
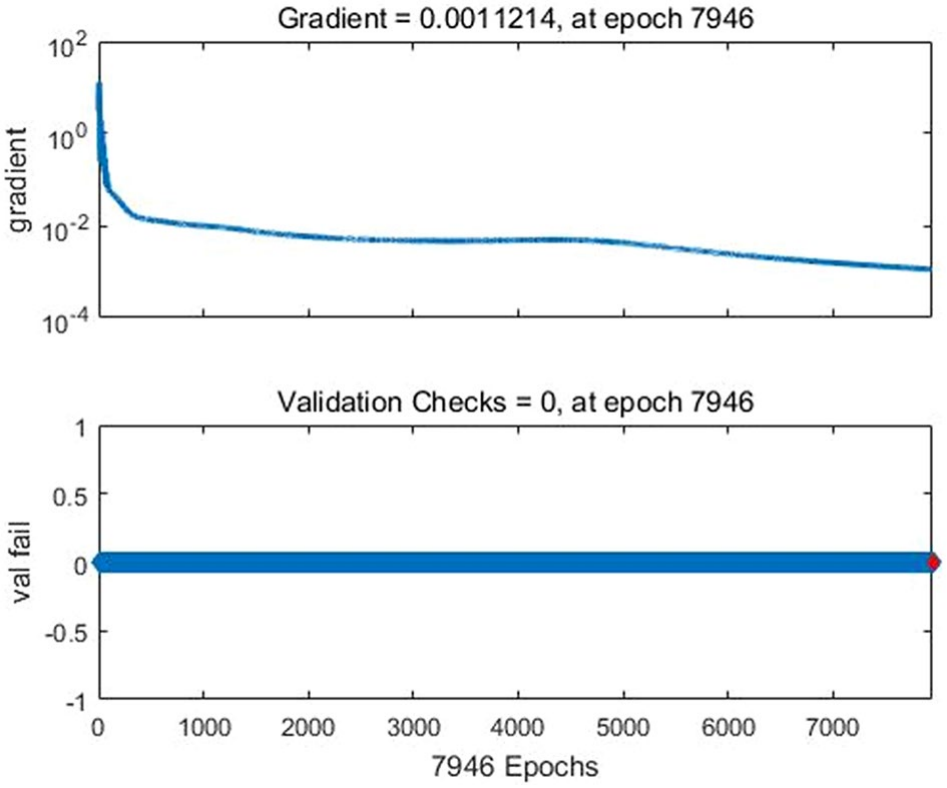
Training parameters of neural network.

**Figure 4:**
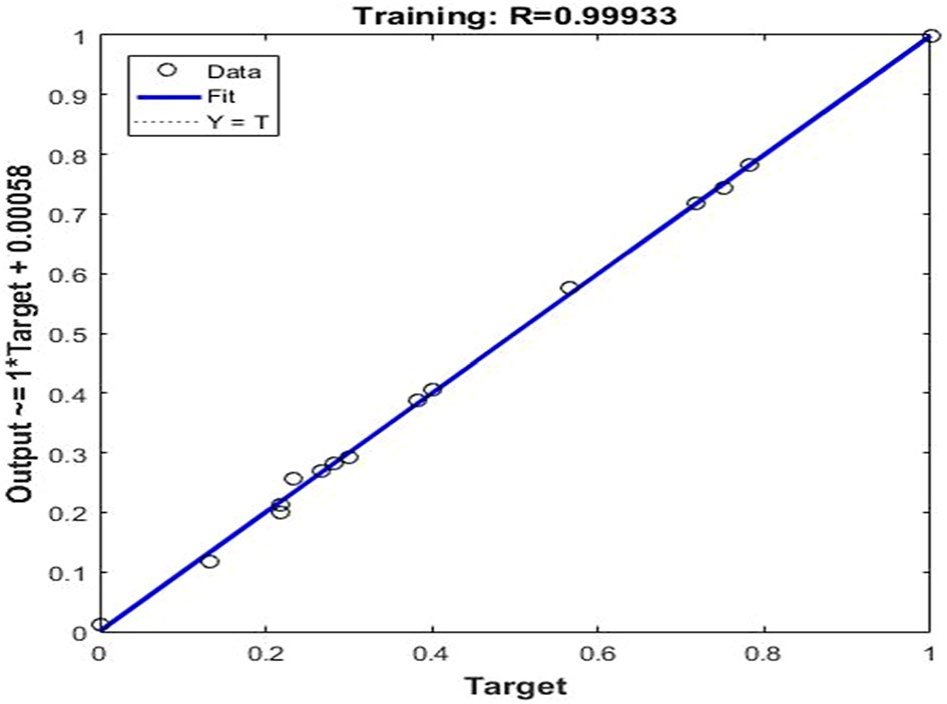
Regression analysis of neural network training.

First, the BP neural network was trained:*net* = *newff(minmax(p),*^1,7^*,{'tansig','purelin'},'traingdm')*. The neural network structure was set as a three-layer neural network of 42–7-1. The learning rate was set to 0.05, the target error was set to 0.0001, and the learning was completed after 7946 iterations of training(Fig. 2). Figures 3 and 4 show that the mean square error was close to 1, indicating that the neural network fit well with the training data. The network after training could therefore better predict the training data.

Second, the training data of each of the independent variables were set to increase10% or decrease 10% to provide two new training data for the independent variables. Using these data for prediction, two groups of results—B_1_ and B_2_—were obtained. The difference between B_1_ and B_2_ was determined, which was called the impact value (IV). Then, an average was taken and denoted mean impact value (MIV). The MIV value of each independent variable was calculated in turn to determine the corresponding influence degree.

### Screening results of evaluation indices based on the MIV-BP model

The MIV of each index related to water resource security in the karst area were sorted by absolute value (Table 2).

**Table 2:** Mean impact values (MIVs) of the influencing factors of water resource security in the karst area.

Index	MIV	Order
*X*_20_ Satisfaction rate of farmland irrigation facilities (%)	− 0.0218	1
*X*_35_ Degree of development and utilization of groundwater (%)	− 0.0212	2
*X*_32_ Proportion of flood and drought losses in GDP (%)	− 0.0199	3
*X*_8_ Fertilizer use per unit of cultivated land (kg/hm^2^ a)	0.0191	4
*X*_40_ Water consumption per ten thousand yuan industrial output (m^3^/ten thousand yuan)	− 0.0161	5
*X*_33_ Urbanization rate (%)	− 0.016	6
*X*_18_ Proportion of water supply for water lifting and diversion projects (%)	− 0.0151	7
*X*_19_ Storage rate of large and medium reservoirs ((%)	0.015	8
*X*_13_ Surface water resources per unit area (10^4^ m^3^/km^2^)	0.0147	9
*X*_26_ Number of karst disasters (times)	0.0138	10
*X*_36_ Degree of development and utilization of surface water (%)	0.0135	11
*X*_9_ Total discharge of waste water (million tons)	0.0125	12
*X*_25_ Soil erosion area ratio (%)	− 0.0116	13
*X*_6_ Qualifying rate of industrial waste water (%)	− 0.0111	14
*X*_42_ Irrigation water consumption per unit area (m^3^/ha)	− 0.0107	15
*X*_21_ Density of large and medium-sized reservoirs (per10^4^ km^2^)	− 0.0106	16
*X*_4_ Number of water pollution accidents (times)	0.0092	17
*X*_37_ Groundwater supply ratio (%)	0.0091	18
*X*_30_ Ecological water use rate (%)	− 0.009	19
*X*_1_ Treatment rate of urban waste water (%)	− 0.0089	20
*X*_3_ Qualifying rate of water environment function zones (%)	− 0.0086	21
*X*_23_ Ratio of effective irrigation area to cultivated land area (%)	− 0.0084	22
*X*_16_ Water production modulus (10^4^ m^3^/km^2^)	− 0.008	23
*X*_29_ The ratio of moderate or severe rocky desertification area (%)	− 0.0079	24
*X*_34_ Water use efficiency (%)	− 0.0078	25
*X*_41_ Water consumption per ten thousand yuan agricultural output value (m^3^/ten thousand yuan)	− 0.0072	26
*X*_12_ Water resources per capita (m^3^/person)	0.007	27
*X*_10_ Average annual rainfall (mm)	0.0068	28
*X*_11_ Groundwater resource per unit area (10^4^ m^3^/km^2^)	0.0066	29
*X*_17_ Water penetration rate (%)	− 0.0066	30
*X*_39_ Water consumption per ten thousand yuan GDP (m^3^/ten thousand yuan)	0.006	31
*X*_31_ Per capita food production (kg)	0.0051	32
*X*_38_ Agricultural water use rate (%)	− 0.0044	33
*X*_14_ Runoff depth (mm)	0.0039	34
*X*_2_ Qualifying rate of the water quality of rivers (%)	− 0.0038	35
*X*_28_ Vegetation coverage (%)	− 0.0037	36
*X*_5_ Discharge intensity of COD in industrial waste water (10^4^t)	0.0035	37
*X*_22_ Ratio of environment and water conservancy personnel (%)	− 0.002	38
*X*_15_ Difference in water volume between entry and exit (10^8^ m^3^)	0.00097	39
*X*_24_ Water investment as a proportion of GDP (%)	− 0.00076	40
*X*_7_ Comprehensive utilization rate of industrial solid waste (%)	− 0.00009	41
*X*_27_ Sediment transport modulus (t/km^2^)	− 0.00001	42

For MIV values, a positive number indicates a positive effect on increased water resource security deviation; a negative number indicates the opposite.

### Grade classification of water resource security

According to Table 2, the index system for water resource security—which includes 31 indices—was established based on indices with MIVs greater than 0.006.

The evaluation index division grade standard for water resource security refers mainly to existing research results and national standards^8,33,34^, including: Environmental Quality Standards for Surface Water[State Environmental Protection Administration 2002 (GB3838-2002)], National Standards for Ecological Demonstration Zones (Trial) [State Ministry of environmental protection (2013)58], Environmental pollution emission standard of Guizhou Province (DB52/864–2013). The indices were divided into five levels, a severe warning level, moderate warning level, critical safety level, moderate safety level, and safety level, which are marked I, II, III, IV, and V, respectively (Table 3).

**Table 3:** Grade classification of water resource security in the karst area.

Target	Index	Grade				
		Severe warning level I	Moderate warning level II	Critical safety level III	Moderate safety level IV	Safety level V
Water quality	C_1_ Treatment rate of urban waste water (%)	< 45	45–60	60–70	70–80	> 80
	C_2_ Qualifying rate of water environment function zones (%)	< 50	50–60	60–70	70–80	> 80
	C_3_ Number of water pollution accidents (times)	> 15	10–15	6–10	2–6	< 2
	C_4_ Qualifying rate of industrial waste water (%)	< 60	60–70	70–80	80–90	> 90
	C_5_ Fertilizer use per unit of cultivated land (kg/hm^2^ a)	> 500	400–500	300–400	200–300	< 200
	C_6_ Total discharge of waste water (million tons)	> 10	7–10	4–7	1–4	< 1
Water quantity	C_7_ Average annual rainfall (mm)	< 300	300–800	800–1200	1200–2000	> 2000
	C_8_ Groundwater resource per unit area (10^4^ m^3^/km^2^)	< 5	5–10	10–20	20–50	> 50
	C_9_ Water resources per capita (m^3^/person)	< 500	500–1500	1500–2,500	2500–3000	> 3000
	C_10_ Surface water resources per unit area (10^4^ m^3^/km^2^)	< 50	50–100	100–150	150–200	> 200
	C_11_ Water production modulus (10^4^ m^3^/km^2^)	< 0.2	0.2–0.4	0.4–0.6	0.6–0.8	> 0.8
	C_12_ Water penetration rate (%)	< 60	60–70	70–80	80–90	> 90
Engineering water shortage	C_13_ Proportion of water supply for water lifting and diversion projects (%)	< 60	60–70	70–80	80–90	> 90
	C_14_ Storage rate of large and medium reservoirs (%)	< 50	50–65	65–70	70–85	> 85
	C_15_ Satisfaction rate of farmland irrigation facilities (%)	< 20	20–40	40–60	60–80	> 80
	C_16_ Density of large and medium-sized reservoirs (per/10^4^ km^2^)	< 2	2–4	4–6	6–8	> 8
	C_17_ Ratio of effective irrigation area to cultivated land area (%)	< 30	30–40	40–50	50–60	> 60
Water resource vulnerability	C_18_ Soil erosion area ratio (%)	> 50	30–50	20–30	10–20	< 10
	C_19_ Number of karst disasters (times)	> 800	600–800	400–600	200–400	< 200
	C_20_ The ratio of moderate or severe rocky desertification area (%)	> 40	30–40	20–30	10–20	< 10
	C_21_ Ecological water use rate (%)	< 1	1–2	2–3	3–5	> 5
	C_22_ Flood and drought losses as a proportion of GDP (%)	> 5.5	4–5.5	2.5–4	1–2.5	< 1
	C_23_ Urbanization rate (%)	< 25	25–35	35–50	50–60	> 60
Water resources carrying capacity	C_24_ Water use efficiency (%)	> 50	30–50	20–30	10–20	< 10
	C_25_ Degree of development and utilization of groundwater (%)	> 10	7.5–10	5–7.5	2.5–5	< 2.5
	C_26_ Degree of development and utilization of surface water (%)	> 50	35–50	20–35	10–20	< 10
	C_27_ Groundwater supply ratio (%)	> 20	15–20	10–15	5–10	< 5
	C_28_ Water consumption per ten thousand yuan GDP (m^3^/ten thousand yuan)	> 400	300–400	200–300	100–200	< 100
	C_29_ Water consumption per ten thousand yuan industrial output (m^3^/ten thousand yuan)	> 320	220–320	120–220	20–120	< 20
	C_30_ Water consumption per ten thousand yuan agricultural output value (m^3^/ten thousand yuan)	> 2000	1500–2000	1000–1500	500–1000	< 500
	C_31_ Irrigation water consumption per unit area (m^3^/ha)	> 1300	900–1300	600–900	300–600	< 300

### Data sources and processing

Primary data were collected from the Guizhou Water Resources Bulletin (2001–2015),water and soil loss in Guizhou Province, the Guizhou Province Environmental Status Bulletin (2001–2015), the Guizhou National Economic and Social Development Statistical Bulletin (2001–2015), and the Guizhou Statistical Yearbook (2001–2015). The data of the indices were normalized by $x_{ij} = \frac{{X_{ij} - X_{\min } }}{{X_{\max } - X_{\min } }}$, where *X*_max_ represents the maximum values of the data, *X*_min_ represents the minimum values of the data, *x*_*ij*_ is the transformed data, and *X*_ij_ is the initial data.

## Model construction

### Construction of the BP neural network

Based on the test results of network structures with different numbers of hidden nodes, a BP neural network with a 31-12-1 structure was established, which involved 31 input nodes, 12 hidden nodes, and one output node. Each sample was represented by annual data, the number of samples was *i* = 1, 2…15, and the number of indices was *j* = 1, 2…31.

For BP network training, the activation function was “logistic.” The BP neural network learning rate was set to 0.6, momentum was set to 0.1, and the maximum number of iterations was 10,000, with iteration until convergence. The performance of the model was evaluated by the mean square error (MSE) of the model test set; a smaller MSE indicates better performance.

### Improvement of the BP neural network

To avoid falling into the local minimum point and fasten the convergence speed, the GA-BP neural network algorithm is applied in this work. On the one hand, GA's search has a good performance in global search, and it is applied to optimize original weights and thresholds of BP neural network. On the other hand, with the more effectiveness in the local search process, BP neural network is trained by the input samples, and once the permissible error is satisfied, the final weights and thresholds are determined. Therefore, GA-BP algorithm integrates their respective advantages and the evaluation veracity is improved to a certain extent. Therefore, with a population size of 20, a mutation probability of 0.1, and 10,000 iterations, the obtained network was transmitted to the BP neural network for further training:

- ga = GA(ds.evaluateModule MSE, net_ga, minimize = True, population Size = 20,
- top Proportion = 0.2, elitism = False, elite Proportion = 0.25, mutation Prob = 0.1,
- mutation Std Dev = 0.2, tournament = False, tournament Size = 2).

The 31 indices of the neural network were divided into five levels, and 10 samples were randomly taken in each level, for a total of 50 (10 × 5) samples. Then, 80% of the data were taken to train the BP neural network, and the remaining 20% of the data were used as a test set. Compare the result with the actual value so as to determine the effect of network category prediction. The outputs of the samples at each level were set as follows: severe warning level (0–0.8), moderate warning level (0.8–1.6), critical safety level (1.6–2.4), moderate safety level (2.4–3.2), and safety level (3.2–4). The training samples were input into the established GA-BP neural network for repeated training. After 10,000 error iterations, the error was 0.0027, which met the error requirements. The training results of the GA-BP neural network were thus reliable and could be put into use.

## Results

### An analysis of the affecting factors about water resource security in the karst area

#### Main factors

 A greater absolute MIV indicates greater the impact on water resource security in the karst area, whereas a smaller value indicates a smaller effect. Table 2 shows that the main factors (MIV greater than 0.01) influencing water resource security were the qualifying rate of industrial waste water, fertilizer use per unit of cultivated land, and total discharge of waste water for the water quality subsystem; surface water resources per unit area for the water quantity subsystem; the proportion of water supply for water lifting and diversion projects, density of large and medium-sized reservoirs, satisfaction rate of farmland irrigation facilities, and storage rate of large and medium reservoirs for the engineering water shortage subsystem; the soil erosion area ratio, number of karst disasters, flood and drought losses as a proportion of GDP, and urbanization rate for the water resource vulnerability subsystem; and the degree of development and utilization of groundwater, degree of development and utilization of surface water, water consumption per ten thousand yuan industrial output, and irrigation water consumption per unit area for the water resource carrying capacity subsystem. The engineering water shortage subsystem therefore had the greatest influence on water resource security in the karst area, followed by the water resource carrying capacity subsystem and the water resource vulnerability subsystem, whereas the water quality and water quantity subsystems had the least influence.

#### Obstruction factors

**Figure 5:**
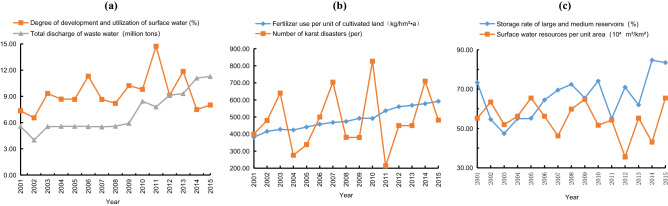
Obstacles to water resource security in the karst area.

 For MIVs, a positive value indicates a positive effect on the increase of water resource security deviation. Table 2 shows that obstacles to water resource security included fertilizer use per unit of cultivated land, storage rate of large and medium reservoirs, surface water resources per unit area, number of karst disasters, degree of development and utilization of surface water, and total discharge of waste water. For each additional standard deviation unit, the deviations of water resource security were 0.0191, 0.015, 0.0147, 0.0138, 0.0135, and 0.0125, respectively. These six indices represent the main obstacles to water resource security in recent years. Figure 5 shows that these six indices are not conducive to water resource security.

Fertilizer use per unit of cultivated land increased year by year, from 381.75 kg/hm^2^ a in 2001 to 591.9 kg/hm^2^ a in 2015. The excessive use of fertilizer in cultivated land can easily cause water pollution. Total discharge of waste water rose from 0.557 billion tons in 2001 to 1.128 billion tons in 2015, which has had an increasing negative impact on water security. The number of karst disasters, degree of development and utilization of surface water, storage rate of large and medium reservoirs, and surface water resources per unit area fluctuated greatly or lingered at a lower level, which put some pressure on the development and utilization of water resource. Therefore, the main factors restricting water resource security in the study area were water pollution caused by agricultural cultivation methods, the method and efficiency of surface water development and utilization, and the storage capacity of water conservancy projects.

#### Driving factors

 Table 2 shows that the driving factors of water resource security in the Guizhou karst area were as follows: the satisfaction rate of farmland irrigation facilities, degree of development and utilization of groundwater, flood and drought losses as a proportion of GDP, water consumption per ten thousand yuan industrial output, urbanization rate, proportion of water supply for water lifting and diversion projects, soil erosion area ratio, qualifying rate of industrial waste water, irrigation water consumption per unit area, and density of large and medium-sized reservoirs. For a decrease or increase of 0.1 standard deviation units, the contributions to reducing the degree of deviation were 0.0218, 0.0212, 0.0199, 0.0161, 0.016, 0.0151, 0.0116, 0.0111, 0.0107, and 0.0106, respectively.

**Figure 6:**
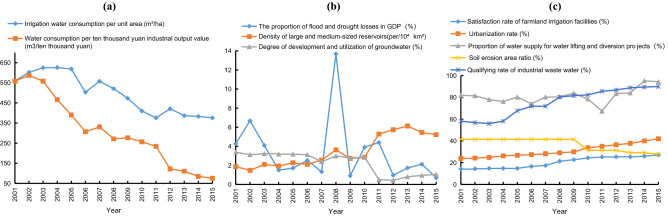
Driving factors of water resource security in the karst area.

According to Fig. 6, from 2001 to 2015 in the Guizhou karst area, flood and drought losses as a proportion of GDP decreased from4.27% to 0.7%, except in 2008 when the value was 13.68%. The irrigation water consumption per unit area, water consumption per ten thousand yuan industrial output, and soil erosion area ratio decreased year by year. In contrast, the satisfaction rate of farmland irrigation facilities, urbanization rate, and qualifying rate of industrial waste water increased year by year. The proportion of water supply for water lifting and diversion projects rose to 94.35% with slight fluctuations. The density of large and medium-sized reservoirs increased from1.87 per/10^4^ km^2^ to 5.22 per/10^4^ km^2^. The degree of development and utilization of ground water decreased to 1.06% in 2015.

### Results of water resource security assessment in the karst area

All index data were normalized and input into the trained GA-BP neural network for testing. The output results are shown in Table 4.

**Table 4:** Results of water resource security evaluation of the karst area based on the GA-BP model.

Order	Year	GA-BP model output value	Evaluation grade
1	2001	1.5935	II
2	2002	1.5654	II
3	2003	1.2889	II
4	2004	1.4308	II
5	2005	1.5976	II
6	2006	1.5882	II
7	2007	1.7027	III
8	2008	1.8760	III
9	2009	1.7870	III
10	2010	1.8125	III
11	2011	1.5768	II
12	2012	2.0831	III
13	2013	1.6648	III
14	2014	2.0533	III
15	2015	2.1509	III

The results in Table 4 show that the water resource security improved from 2001 to 2015. The water resource security in the Guizhou karst area was at a moderate warning level from 2001 to 2006, with a steady increasing trend. From 2007 to 2015 (except 2011), the safety level was critical, but there were slight decreases in 2009 and 2013. The safety level deteriorated to a moderate warning level in 2011. During this period, the deteriorating situation of water resource security has been alleviated.

## Discussion

### Solving the problem of engineering water shortage is key to ensure water resource security in the karst area

 It can be seen from the subsystems of the indices sorted by the absolute MIV that the engineering water shortage subsystem had the greatest impact on water resource security in the karst area, which is the main reason to promote its transformation.

The water shortage in karst areas is caused by poor natural conditions and inadequate engineering conditions, that is, “engineering water shortage”. It is a serious problem in the Guizhou karst area. The main reasons are as follows. First, the karst hydrogeological and geomorphic conditions, with high mountains and deep rivers, make Guizhou a water shortage area. Second, the karst area is rich in water resources, but it is difficult to develop and utilize these resources. Inter annual variations of rainfall are not significant, but there are large differences within the year, which can easily lead to seasonal drought. Third, the layout of water conservancy projects such as water retention, water storage, and water transfer is unreasonable or insufficient, resulting in conditions of shortage of irrigation and the inadequacy of drinking water for people and livestock. Therefore, the Guizhou karst area has become an area of water shortage, especially engineering water shortage. This is the main bottleneck restricting the coordinated development of the region’s social economy and ecology.

Water conservancy projects can determine the diversion and allocation of water resources across time and district to achieve reasonable allocation, efficient utilization, and protection. This indicates the need for higher requirements for engineering water storage and improving water resource utilization efficiency. Therefore, the construction of water conservancy projects is key to ensure future water resource security.

### The modes of development and utilization of water resources are also significant in the karst area

 In the past 15 years, Guizhou Province has attached great importance to the development and utilization of water resources. The subsystems of water resource carrying capacity and vulnerability in the Guizhou karst area have risen steadily, which has improved water resource security. However, the development and utilization of water resources will cause changes in the quantity and structure of water usage. This has both optimization and constraints on regional development. Therefore, the geological, hydrological, and hydrogeological characteristics of the karst area must be investigated. The development and utilization of water resources in the karst area should involve appropriate technologies or methods in accordance with these different hydrogeological structures. Geology, geomorphology, rainwater, distributions of farmland and residences, and hydrogeological structures in the karst area are the major factors to consider for solving water shortages in this area^35^. Rain collection, underground reservoirs, a decentralized water supply and runoff gathering are significant modes of development in the karst area.

### The situation of water resource security in karst area of Guizhou is gradually getting better

 This is achieved through water conservation projects and technological measures for water resource exploitation, utilization, projection, and reasonable allocation and control. Meanwhile, Guizhou achieves the security of regional water resource utilization and development through adjusting the regional economic pattern, water resource utilization technology, and so forth.

From 2001 to 2006, the status of water resource security was serious, and there was a moderate warning level. At that time, the industrialization of Guizhou province was developing rapidly, and the construction of water conservancy and other infrastructure was also advancing rapidly. Increased attention was given to soil erosion, desertification, water resource pollution, and other problems. Despite high water consumption, the water environment was gradually improving. However, rapid economic and social development has exceeded the carrying capacity of the water resources during this period. Some problems persist in the study area, such as inadequacy of urban sewage treatment facilities, outdated water conservancy facilities, and insufficient prevention of environmental pollution. Urban water pollution treatment facilities and garbage treatment facilities are seriously outdated and cannot meet the requirements of urban development and water environmental protection. These problems have led to a low starting point for water resource security utilization in Guizhou Province. Although the situation has been improved and alleviated year by year, it is still in a moderate warning level, and the water resource security situation is still severe.

After reaching the critical safety level in 2007, the water resource security of Guizhou Province declined slightly in 2009 and 2013, although a critical safety level was maintained; the safety level further deteriorated to a moderate warning level in 2011. This deterioration occurred because Guizhou suffered its worst drought in a century from 2009 to 2011, and another drought in 2013. According to the information provided by single indices, the treatment rate of urban waste water, proportion of water supply for water lifting and diversion projects, qualifying rate of water environment function zones, qualifying rate of industrial waste water, degree of development and utilization of groundwater, and density of large and medium-sized reservoirs all showed increasing trends year by year or showed relatively high levels. In contrast, the indices of irrigation water consumption per unit area, above moderate rocky desertification area ratio, water consumption per ten thousand yuan GDP, and water consumption per ten thousand yuan industrial output decreased year by year. All of these indices played a driving role in water utilization and water resource security in the study area. Although the once-in-a-century drought reduced the amount of water, Guizhou Province improved the utilization rate of water resources in the dry years, which alleviated the impact of the reduction of water resources to a certain extent, and allowed the water resource security in the study area to barely maintain the critical safety level. This finding is consistent with previous research conclusions: the engineering water shortage subsystem had largest effect on water resource security in the karst area, whereas the water quantity subsystem had the least influence.

It can be inferred that the requirements for ensuring water resource security in the karst area are a good economic development model, environmental protection, pollution control, and improvement of basic water conservancy facilities. These measures can be conducive to actively coping with the impact of abnormal climate changes on the utilization of water resources.

## Conclusions

The research presented in this paper highlights the assessment of water resource security in a karst area by an ANN. Assessment of water resource security is usually based on the balance of economic, social, and environmental systems. In karst areas, water resource security assessment is difficult because of the complexity and uncertainty of the water resource systems. Therefore, a three-layer ANN model was used to understand the complex relationship of the karst water security level and its influencing factors.

The MIV-BP neural network was built in this work to assess the main influencing factors of water resource security based on analysis of the water quality subsystem, water quantity subsystem, engineering water shortage subsystem, water resource vulnerability subsystem, and water resource carrying capacity subsystem. In addition, the GA-BP neural network was established to assess water resource security from 2001 to 2015.The evolution of water resource security with time was analyzed. The results show that the water resource security in the Guizhou karst area has generally followed a rising trend. For protection and management of water resources in a karst area, the modes of development and utilization of water resource must be thoroughly understood.

This work provides a theoretical reference for promoting the utilization of water resources in the Guizhou karst area and a method for assessment of water resource security. The results can provide an important foundation for water management in the Guizhou karst area.
